# Profile of idiopathic parkinson’s disease in Moroccan patients

**DOI:** 10.1186/1755-7682-7-10

**Published:** 2014-03-06

**Authors:** Wafa Regragui, Lamiae Lachhab, Rachid Razine, Leila Raissouni, Khaoula Rasmouni, Fatima Imounan, El hachmia Ait Benhaddou, Redouane Abouqal, Ali Benomar, Mohamed Yahyaoui

**Affiliations:** 1 Department of Neurology B and Neurogenetics, Hôpital des Spécialités O.N.O, 6444 Rabat, Morocco; 2 Laboratory of biostatistics, clinical research and epidemiology, Faculty of medicine and pharmacy of Rabat, Rabat, Morocco; 3 Centre de recherche en épidémiologie clinique et essais thérapeutiques Faculty of medicine and pharmacy of Rabat, Rabat, Morocco; 4 Department of Public Health, Faculty of medicine and pharmacy of Rabat, Rabat, Morocco; 5 University Mohamed V Souissi, Angle avenue Allal El Fassi et Mfadel Cherkaoui, Al Irfane, Rabat, Morocco

**Keywords:** Parkinson’s disease, Clinical features, Treatment, Motor complications, On-motor complications

## Abstract

**Objective:**

To characterize clinical aspects of Idiopathic Parkinson’s disease from a movement disorders consultation in University Hospital of Rabat.

**Methods:**

Retrospective review of medical records of 117 patients with diagnosis of Idiopathic Parkinson’s disease seen in our outpatient clinic from 2006 to 2011.

**Results:**

Mean age was 64 ± 10 years with predominance of men (61.5%). Mean age at disease onset was 57 ± 11 years. Early onset Parkinson’s Disease was recorded in 12.8%. The median duration of disease was 5 years. Initial symptom appeared on the right side in 56.5%. Tremor presentation was the most frequent (40.2%). Symptom severity was mild to moderate in 80% of cases (UPDRS < 30). Forty four per cent of patients were receiving both Dopamine Agonists and Levodopa and in 69% of cases Levodopa was introduced within the first year following onset. The mean Levodopa Equivalent Doses (LED) was 667 ± 446 mg/day. Motor complications were found in 42% with motor fluctuations in 28.7% and ^2^dyskinesias in 26.7%. Non motor complications are represented mainly by autonomic disorders (44%). There were no differences in the clinical presentation related to the age at onset. Age of onset < 45 and LED > 600 mg are identified as risk factors for motor fluctuations whereas duration of Levodopa treatment is a risk factor of dyskinesias.

**Conclusion:**

Our patients are younger compared to most series with high prevalence of early onset forms. In the majority of cases, Levodopa was introduced within the first year following onset which expose patients to dyskinesias early in the course of the disease.

## Introduction

Parkinson’s disease (PD) is the second most common neurodegenerative disorder. In general population, its prevalence varies between 18 and 234 cases/100000 people [1].

The mean age of onset is 58 to 62 years and early onset PD, before 40 years, is rare (< 10%) [2]. Symptoms of PD are due to a progressive loss of nigral neurons causing striatal dopaminergic denervation. The advent of levodopa therapy has improved the quality of care but the evolution is characterized by motor complications in 75-80% of cases after 5 years, they are represented mainly by dyskinesias and motor fluctuations [3,4]. In addition, the health-related quality of life of PD patients is related not only to their motor disability, but also to their non-motor symptoms of depression, sleep disturbance, bladder and sexual dysfunction.

To our knowledge, there are no studies reporting epidemiological and clinical features of PD in Morocco. Our study tries to give an overview of the clinical presentation and management of Idiopathic Parkinson’s disease in Moroccan people, from a movement disorders consultation in Ibn Sina University Hospital of Rabat.

## Methods

Our series is a study of medical records of 117 patients with diagnosis of Idiopathic Parkinson’s disease consecutively seen in our outpatient clinic from January 2006 to January 2011. The patients recruited are representative of Moroccan population as the university hospital of Rabat is the first one in Morocco and patients are mainly from the north-west of the country. The patients were recruited based on the Queen Square Brain Bank Clinical Criteria at clinical assessment [5]. They were examined by the same neurologist. Their clinical records were analyzed, with a farm which specifies the demographic characteristics (age, gender, age of onset, duration of the disease), clinical features (laterality of symptoms, predominant feature: Tremulous or akinetic-rigid or mixed, disease severity assessed by UPDRS motor score (Unified Parkinson’s Disease Rating Scale) and Hoehn and Yahr score [6], motor complications (fluctuation, dyskinesia, freezing) and non-motor complications (dysautonomic troubles, sleep disorders, depression, cognitive disturbance, hallucination, delirium), treatment regimen (Levodopa treatment, dopaminergic agonist treatment, Levodopa-agonist combination, anticholinergic treatment), mean Levodopa Equivalent Doses (LED) which expresses different doses of anti-parkinsonian medications on a single scale calculated using Deuschl et al.’s method [7], delay of Levodopa introduction and duration of L-dopa treatment.

### Statistical analysis

Categorical variables were expressed as effectifs and percentages and continuous variables were expressed as means ± SD or median (interquartile range). The 95% confidence intervals (CIs) were estimated. To study risk factors, univariate analyses were first performed using simple logistic regression. Variables with p < 0.20 in the univariate analysis were tested in the multivariate analysis. Adjusted odds ratios (OR) and their 95% CIs were derived. A *p* value of 0.05 or less was considered to be statistically significant. Data were analyzed using the statistical soft-ware SPSS version 13.0.

## Results

### Demographic characteristics

Mean age of patients was 64 ± 10 years (range 29–85) with masculine predominance (61.5%) and a sex ratio of M/F = 1.6. Mean age of disease onset was 57.5 ± 11 (range 28–80) years and 13.2% of patients presented an early onset PD with onset before 45 years. Median of disease duration was 5 years (range 3–9).

### Clinical presentation

Initial symptoms’ side onset was right in 56.5% (65) of patients, left in 31.3% (36) and bilateral but asymmetrical in 12.2% (14). The predominant phenotype was tremulous in 40.2% (47) of patients, akineto-rigid in 28.2% (33) and mixed in 31.6% (37). Mean motor UPDRS score was 20.5+/−14.5 (0–75). Symptom severity, as measured by the UPDRS score, was mild to moderate in 80% (UPDRS <30) and mean Hoehn and Yahr score was 1.77+/−0.7 (1–3).

There were no differences in the clinical presentation related to the age at onset.

### Treatment

Forty four per cent (48) of patients were receiving both dopamine agonists and Levodopa whereas 37.7% (41) received only Levodopa and 18.3% (20) received only dopaminergic agonists.

Anticholinergic drugs were used in 22.3% (26) of patients. The delay of levodopa treatment was 11.6 months (0–120) and 68% (74) of patients received L-dopa as first treatment with mean duration of exposition of 5 years (3–9). In 69% of cases, Levodopa was introduced within the first year following onset. The mean Levodopa Equivalent Doses (LED) was 66 ± 446 mg/day (0–2212).

### Motor complications

Motor complications were found in 42% (48) of our patients. Motor fluctuations appeared in 28.7% (33) of patients after 3.7 years of disease duration, dyskinesias in 26.7% (31) after 5.5 years of evolution, freezing in 17.2% (20) and falls in 4.3% (5) of patients.

#### Motor fluctuations

On univariate analysis, age of onset <45 years (p < 0,001; OR = 35,7; IC = [5,6-224]), duration of the disease (p = 0.019; OR = 1.1; IC = [1.01-1.21], LED >600 mg/j (p < 0.001; OR = 9.55; IC = [3.6-25.3]) and association of levodopa and dopaminergic agonists ( p < 0.001; OR = 8.5; IC = [1.7-40.8]) are the factors associated to the occurrence of motor fluctuations. However, on multivariate analysis, only age of onset and LED were associated to fluctuations. Patients with age of onset <45 develop fluctuations 40 times more comparing to an age > 65 (p = 0.002, OR = 40, IC [3.9- 410]) and motor fluctuations were 4 times more frequent in patients with LED > 600 mg (p = 0.03; IC [1.1- 13.9]) (Table 1).

**Table 1:** Fluctuations associated factors

**Independant variables**	**n (%)**	**Univariate analysis**			**Multivariate analysis**		
		**OR**	**CI 95%**	***p***	**OR**	**CI 95%**	***P***
Age of onset							
> *65 years*	2 (7,1)	1					
< *45 years*	11 (73,3)	35,7	5,6 - 224	<0,001	40	3,9- 410	0,002
*45*-*54 years*	11 (40,7)	8,90	1,75 - 45	0,008	5,80	0,92 - 37	0,06
*55*- *65 years*	8 (19,0)	3,05	0,5 - 15,6	0,17	2,10	0,36-13,1	0,39
Gender							
*Men*	22 (31,0)						
*Women*	11 (25,0)	0,70	0,31 - 1,73	0,49			
Disease duration*	5 [3;9]	1,10	1,01 – 1,21	0,019	0,90	0,98-1,008	0,63
Treatment							
*Dopaminergic agonists*	2 (10,5)	1					
*Levodopa*	7 (17,1)	1,70	0,3 – 9,3	0,5	0,17	0,01- 2.36	0,18
*DA* + *levodopa*	24 (50,0)	8,50	1,7 – 40,8	<0,001	2,10	0,63- 7,3	0,22
LED							
≤ *600 mg*/*day*	7 (11,5)						
> *600 mg*/*day*	26 (55,3)	9,55	3,6- 25,3	<0,001	3,94	1,1- 13,9	0,03
L-dopa treatment duration*	5 [3;9]	0,90	0,96- 1,1	0,32			

*median and quartiles ; *n* (%) : *number* (*percentage*) ;*OR* : *odds*-*ratio*; *CI*: *confidence interval*; *LED* : *levodopa equivalent dose*.

#### Dyskinesias

On univariate analysis, age of onset <45 years (p = 0.008; OR = 7.1; IC à 95% = [1.65-30.8]), duration of the disease (p = 0.013; OR = 1.1; IC = [1.02-1.22]), LED > 600 mg/j (p = 0.005; OR = 3.5; IC = [1.46-8.36]), exposition to levodopa duration >5 years (p = 0.009; OR = 3.2; IC = [1.34-7.95]) and delay of levodopa treatment ≤ 3 months (p = 0.04; OR = 2.9; IC = [1.02-17]) were the factors associated to occurrence of dyskinesias. However, considering the same treatment regimen, only early introduction of levodopa increases the risk of dyskinesias by 3.2 with p = 0.03 on multivariate analysis using descending method (Table 2).

**Table 2:** Dyskinesias associated factors

**Independant variables**	**n (%)**	**Univariate analysis**			**Multivariate analysis**		
		**OR**	**CI 95%**	***p***	**OR**	**CI 95%**	***p***
Age of onset							
> *65 years*	4 (13,8)	1					
< *45 years*	8 (53,3)	7,1	1,65 – 30,8	0,008			
*45*-*54 years*	8 (29,6)	2,6	0,68 - 10	0,15			
*55*- *65 years*	11 (26,2)	2,2	0,62 – 7,8	0,21			
Gender							
*Men*	22 (30,6)						
*Women*	9 (20,5)	0,58	0,24 - 1,42	0,2			
Disease duration*	5 [3;9]	1,1	1,02 - 1,22	0,013			
Hoehn and Yahr score							
≤ *2*	5 (20,0)	1					
> *2*	20 (26,7)	1,4	0,49 - 4,39	0,5			
Treatment							
*Dopaminergic agonists*	2 (10,5)	1			1		
*Levodopa*	9 (22,0)	0,41	0,08 - 2,15	0,29	0,4	0,15 -1,06	0,06
*DA* + *levodopa*	20 (41,7)	2,5	0,99 - 6,47	0,051	0,14	0,01-1,28	0,08
LED							
≤ *600 mg*/*day*	11 (17,5)	1					
> *600 mg*/*day*	20 (42,6)	3,5	1,46- 8,36	0,005			
L-dopa treatment duration*	5 [3 ;9]	1,01	1,002 - 1,01	0,014			
≤*5 years*	60 (59,4)						
>*5 years*	41 (40,6)	3,27	1,34- 7,95	0,009			
Délai de début de L-dopa							
>*3 months*	5 (15,6)	1					
≤ *3 months*	25 (35,7)	2, 9	1,02- 17,0	0,045	3,2	1,07-9,71	0,03

*median and quartiles ; *n* (%): *number* (*percentage*); *OR* : *odds*-*ratio*; *CI*: *confidence interval*; *LED: levodopa equivalent dose*.

#### Freezing

Freezing is correlated to duration of Levodopa therapy (p = 0.008, OR = 1.01, IC = [1.003-1.02]) and LED >600 mg (p = 0.002). On multivariate analysis, only LED >600 mg is highly associated to freezing (p = 0.006; OR = 4.8; IC = [1.5-14.9]) (Table 3).

**Table 3:** Freezing associated factors

**Independant variables**	**n (%)**	**Univariate analysis**			**Multivariate analysis**		
		**OR**	**CI 95%**	***p***	**OR**	**CI 95%**	***p***
Age of onset							
> *65 years*	4 (14,3)	1					
< *45 years*	5 (33,3)	3	0,66- 13,5	0,15			
*45*-*54 years*	5 (17,9)	1,3	0,31- 5,4	0,71			
*55*- *65 years*	6 (14,3)	1	0,25- 3,9	1,00			
Gender	-	0,35	0,10- 1,10	0,078			
Disease duration*	5 [3;9]	1,1	1,03- 1,26	0,009	1	0,99-1,01	0,07
Dysautonomic troubles	13 (26,0)	1,5	0,78- 2,90	0,22			
Cognitive troubles	4 (30,8)	2,3	0,65- 8,70	0,18			
L-dopa treatment duration*	5 [3;9]	1,01	1,003- 1,02	0,008	1	1,1-11,8	0,06
*LED* (mg) **	667 ± 446	1,001	1,000- 1,002	0,02			
≤ *600 mg*	5 (7,8)	1					
> *600 mg*	15(32,6)	0,17	0,05- 0,50	0,002	4.8	1,56-14,9	0,006
Treatment							
*Dopaminergic agonists*	0	1					
*Levodopa*	9 (22,0)	0,92	0,33- 2,50	0,87			
*DA* + *levodopa*	11 (23,4)	0		0,71			

*median and quartiles ;**mean and standard deviation; OR: odds-ratio; CI: confidence interval; LED: levodopa equivalent dose.

#### Non motor complications

Non motor complications consist mostly in dysautonomic troubles in 44% (51) of cases and sleep disorders in 18.1% (21). Depressive symptoms were found in 12% (14) of cases, cognitive disturbance in 11.2% (13), hallucinations in 8.5% (10) and delirium in 2.6% (3) of patients.

There was no association between duration of the disease and dysautonomic troubles (p = 0.9) nor sleep disturbances (p = 0.3). Furthermore, there was no association between hallucinations and LED, medication type, duration of evolution, depression or cognitive troubles.

### Gender phenotype

There were no gender differences in clinical characteristics, demographic features and complications, while there is a difference regarding the Hoehn and Yahr score (≤ 2 in 87.8% of women vs. 65% in men, p = 0.01) (Table 4).

**Table 4:** Gender phenotype

**Characteristics**	**Gender**		***P***
	**Men**	**Women**	
Age of onset*			0,32
< *45 years*	12 (80,0)	3 (20,0)	
*45 to 54 years*	17 (60,0)	11 (39,3)	
*55 to 65 years*	27 (64,3)	15 (35,7)	
> *65 years*	15 (51,7)	14 (48,3)	
Predominant feature*			
*Tremulous*	28 (38,9)	19 (42,2)	0,50
*Akineto*-*rigid*	23 (31,9)	10 (22,2)	
*Mixed*	21 (29,2)	16 (35,6)	
Side onset *			
*Right*	41 (58,6)	24 (53,3)	0,41
*Left*	19 (27,1)	17 (37,8)	
*Bilateral*	10 (14,3)	4 (8,9)	
Hohn and Yahr score*			
≤ *2*	39 (65,0)	36 (87,8)	0,01
>*2*	21 (35,0)	5 (12,2)	
UPDRS score **	21,5 ± 16	19,1 ± 12,6	0,44
Disease duration**	6,99 ± 4,73	6.28 ± 4,31	0,42
Motor fluctuations*	22 (31,0)	11 (25,0)	0,49
Dyskinesias*	22 (30,6)	9 (20,5)	0,23
Freezing*	16 (22,2)	4 (9,1)	0,07
Hallucinations*	9 (12,5)	1 (2,2)	0,08
Dysautonomic troubles*	35 (48,6)	16 (36,4)	0,21
Depression*	9 (12,5)	5 (11,4)	0,85
Cognitive troubles*	8 (11,1)	5 (11,4)	0,96

*number (*percentage*) ;**mean ± *standard deviation*.

## Discussion

This study allowed us to identify some specific features of PD in Moroccan patients.

In fact, our patients were younger compared to other series, 57.5 ± 11 years old versus 61.3 ± 10.9 years old for Lopez et *al*. [8] and 67 ± 11.6 years old for Yust-Katz and *al*[9]. This difference may be explained by high prevalence of early onset PD in our series that suppose a specific genetic profile in our population.

The men predominance is similar to other series [10,8,12]. These findings could suggest a clinical difference between men and women but it hasn’t been investigated in Parkinson’s disease series.

Tremulous phenotype is the most frequent without age intercession as reported by Eun Joo Chung *et al*. [2]. However, Wickremaratchi *et al*. [10], reported an increasing of tremulous phenotypes with age. In fact, studies are discordant concerning the influence of age on the clinical subtype.

The predominance of symptoms on the right side has been noted in our patients and in other series [9]. Schlomit et al. have reported in their series of 307 patients the asymmetry of symptoms and their predominance on the dominant side [9]. Many hypothesis have been proposed to explain this asymmetry such as genetic predisposition, inborn variation of dopaminergic neurons number in one side, environmental, metabolic or toxic factors [13,14].

Most of our patients (69%) received levodopa as initial treatment. The lack of agonists in Morroco, as only piribedil is available, may explain the initial LevoDopa treatment or its early use when piribedil becomes insufficient in controlling symptoms.

Our patients have moderate handicap scores (Hoehn and Yahr ≤2, motor UPDRS < 30). This early use of levodopa with high dose may explain the moderate severity of Hoehn and Yahr score and UPDRS in our patients as they were assessed while taking their medicine. Indeed, our mean LED is 1.5 times the one pointed out by Politis et al. with the same duration of disease [12].

Motor complications are represented by motor fluctuations and dyskinesias. Motor fluctuations occurred after 3.7 years of evolution. Similar findings were reported by Lopez et al. [8] which could be explained by the use of levodopa as first line treatment in 69% of patients. However, duration of levodopa therapy is not a risk factor for motor fluctuations in our study while early onset and LED > 600 mg/j were correlated to fluctuations on multivariate analysis.

In other studies, duration of levodopa therapy and severity of the disease (Hoehn and Yahr ≥2) were recognised as risk factors for fluctuations [15].

Concerning dyskinesias, after five years of levodopa therapy, less than one third of our patients presented dyskinesias. This has also been reported in other series in which the prevalence of dyskinesias is 23% [10] while therapeutic trials data suggest that 40% of patients are at risk of developing dyskinesias after 4 to 6 years of levodopa treatment [16]. This difference could be due to the age of patients in therapeutic trials, the treatment regimen and the scores used to identify dyskinesias. In our series, only inaugural use of Levodopa was a risk factor of dyskinesias as it multiplied the risk by 3. On the other hand, early age of onset, LED >600 mg/j and the duration of disease, typically reported in literature have not been identified as risk factors on multivariate analysis [10].

Freezing was rarely found in our patients (17.2%) comparing to the literature. In ancient publications, prevalence of freezing reached 50% after 5 years of evolution [17,18], while in recent series it varies between 26 to 32% [19,20]. This decrease in the prevalence of freezing is most likely due to the use of dopaminergic agonists that allows lowering the doses of levodopa. In fact, the doses of levodopa and severity of the disease are the risk factors of the occurrence of freezing [19,20] and it was also confirmed in our series as a LED > 600 mg/j was the only risk factor.

Non motor complications are represented mainly by dysautonomic troubles (44%) and weren’t correlated to disease duration which has also been reported by Sandyk and Awerbuch (1992) [21]. Depression was found only in 12% of cases, while it has been reported in 40 to 70% in other series [22,23]. This difference is probably due to an underestimation of depression in our patients especially that no depression scale was used in our study but the special pathology perception in our population can be another explanation as faith could be a factor of a better acceptance of illness in our population.

As quoted in results, women have less severe disease. Miller and al. reported the same findings with more features such as a later age of onset, tremulous phenotype predominance, much severe dyskinesias and more depression for women [11].

Our results are limited by the retrospective character of our study and non use of specific scales (especially for non motor complications). Additionally, the study was led in one center, even if it’s a specialized one. Yet it’s the first Moroccan series describing Parkinson disease and surely a starting point and an experience for other local studies.

## Competing interests

The authors declare that they have no competing interests.

## Authors’ contributions

RW and LL participated equally to the work.RW participated in the design of the study and the acquisition of data, LL and RL draft the manuscript, RK and RL participated in the acquisition of data, IF and RR performed the statistical analysis and interpretation of data, AE, BA and YM participated in the coordination of data, AR gave the final approval of the manuscript. All authors read and approved the final manuscript.
